# Social networks and obesity among Somali immigrants and refugees

**DOI:** 10.1186/s12889-020-8315-7

**Published:** 2020-02-17

**Authors:** Jane W. Njeru, Mark L. Wieland, Janet M. Okamoto, Paul J. Novotny, Margaret K. Breen-Lyles, Ahmed Osman, Yahye A. Ahmed, Mohamud A. Nur, Omar Nur, Irene G. Sia

**Affiliations:** 1 Department of Medicine, Mayo Clinic, Rochester, MN USA; 2 Research Laboratories, Mayo Clinic, Scottsdale, AZ USA; 3 Division of Biomedical Statistics and Informatics, Mayo Clinic, Rochester, MN USA; 4 Gastroenterology Research Unit, Mayo Clinic, Rochester, MN USA; 5 Somali Community Resettlement Services, Rochester, MN USA; 6 Somalia Rebuild Organization, Rochester, MN USA

**Keywords:** Community-based participatory research, Immigrants, Obesity, Refugees, Social network analysis, Somali

## Abstract

**Background:**

Somali immigrants and refugees to the United States are at high risk for obesity and related cardiovascular risk. Social network factors influence health behaviors and are important contributors to the obesity epidemic. The objective of this study was to describe social networks and obesity-related characteristics among adult Somali immigrants in a Minnesota city in order to inform a community-based, participatory, research-derived, social network intervention to decrease obesity rates.

**Methods:**

Survey data (demographics, general health measures, and sociobehavioral and network measures) and height and weight measures (for calculating body mass index) were collected from adult Somali immigrants by bilingual study team members at community locations. Descriptive statistics were used to report the survey and biometric data. Logistic regression models were used to describe the basic associations of participants and network factors. Network data were analyzed to identify nodes and ties, to visualize the network, and to identify potential interventionists for a future social network intervention.

**Results:**

Of the 646 participants, 50% were overweight or affected by obesity. The network had 1703 nodes with 3583 ties between nodes, and modularity was high (0.75). Compared with respondents of normal weight, participants who were overweight or affected by obesity had more network members who were also overweight or obese (odds ratio [OR], 2.90; 95% CI, 1.11–7.56; *P* = .03); this was most notable for men (OR, 4.58; 95% CI, 1.22–17.22; *P* = .02) and suggestive for those 50 years or older (OR, 24.23; 95% CI, 1.55–377.83; *P* = .03). Weight loss intention among participants who were overweight or affected by obesity was associated with number of family members and friends trying to lose weight, enabling functional network factors (social norms for weight loss, social support for healthy eating, and social cohesion), and less favorable obesogenic social norms.

**Conclusions:**

In this community sample of Somali immigrants, distinct social networks are clustered by weight status, and social contacts and functional network characteristics are related to individuals’ weight loss intentions. These factors should be considered in weight loss interventions and programs. A social network intervention targeting weight loss, within a community-based participatory research framework, is feasible in this vulnerable population.

## Background

In the early 1990s, after the outbreak of civil war in their country, Somali refugees began arriving in the United States (US) [1, 2]. They constitute the largest group of refugees from sub-Saharan Africa in the US and a sizable proportion of immigrants from Africa, with their numbers doubling in each of the past 3 decades [3, 4]. Forty percent of Somali refugees settled in Minnesota, where they compose the majority of the 21% of the state’s foreign-born population that is from Africa [5].

Somali immigrants and refugees in the US and other Western countries have disproportionately higher rates of obesity and related cardiovascular disease risks, such as diabetes mellitus [6–12]. The higher rates have been postulated to result from the adoption of lifestyles that include low levels of physical activity and a diet rich in refined carbohydrates and fat [10, 13, 14]. In turn, these lifestyle and behavioral factors that promote the development of obesity are enabled by multiple, complex, and interconnected social and environmental factors [15, 16]. To effectively address this obesity epidemic, interventions that take into account the social context of individuals and communities need to be explored.

Within the general population, social network structures have been identified as contributors to the obesity epidemic [17, 18], in part, through transmission of obesogenic behaviors [19]. It is postulated that structural and functional characteristics of groups (eg, social networks) influence behaviors through several pathways. Structural network factors, such as size, density, and composition, may affect obesity-related behavior through normative self-comparison and thereby direct the influence of peers (among other factors), leading to adoption of similar behaviors. Functional network factors, in contrast, may influence obesity-related behavior through shared social activities, social norms, social cohesion, and social support [20]. The potential role of social network structures in the obesity crisis has been demonstrated in certain immigrant populations, including Hispanic and Eastern European immigrants [21], but not among immigrants and refugees from sub-Saharan Africa.

Social network interventions involve purposeful use of existing social networks in the natural environment to promote positive behavior change and health outcomes [22], and they have been associated with consistent findings of positive behavior change [23]. The understanding of network factors within different populations at risk for obesity (eg, Somali) is an important step toward derivation of a network intervention aimed at attenuating this risk.

Like other minority groups in the US, refugee and immigrant populations are hard to reach through traditional research and intervention pathways, and they may not easily adapt to existing community-based programs and resources [24]. In community-based participatory research (CBPR), community members and academics partner in an equitable relationship through all phases of research and intervention development. CBPR is well suited to address the interplay between behaviors and social determinants of health [25, 26], and it has been an effective means of approaching health topics among immigrant and refugee populations [27].

Rochester Healthy Community Partnership (RHCP) is a CBPR partnership whose mission is “to promote health and well-being among the city population through community-based participatory research, education, and civic engagement to achieve good health for all” [19]. RHCP was conceived in 2004 and has been effective in designing and implementing several interventions with immigrant and refugee populations in Rochester, Minnesota [28–30]. In the Healthy Immigrant Families (HIF) randomized controlled trial, RHCP community and academic partners developed and tested a healthy eating and physical activity intervention among Somali families [28]. The adults in the participating families had significant improvement in dietary quality. Subsequently, through diffusion of the intervention through word of mouth in the community, the RHCP partners conceived of a possible social network intervention, through which a version of the healthy eating and physical activity intervention used in HIF could be modified to be delivered by peer interventionists within the social networks. As a first step, a social network analysis needed to be conducted, and obesity-related network characteristics among Somali refugees and immigrants in the community needed to be described.

The hypotheses of the present study were 1) that an association exists between the weight status of study participants and that of persons in their social network; 2) that for participants who are overweight or obese, an association exists between their weight loss intentions and the intentions of persons in their social networks; and 3) that social norms for weight loss, social support for healthy behaviors, and social cohesion would mediate the relationship between participants weight loss intentions and their social network. The overall goals of this study were 1) to elucidate social networks among Somali immigrants and refugees in Rochester, Minnesota; 2) to describe obesity-related network characteristics; and 3) to identify peer leaders as potential future interventionists within networks. This work will form the foundation of a CBPR-derived social network intervention for nutrition and physical activity that targets behavior change to decrease obesity rates and cardiovascular risk among community members who are overweight or affected by obesity.

## Methods

### Study setting and participants

This study was conducted in a participatory manner, consistent with CBPR norms, by RHCP members and academic and community partners from the Somali community. Participant recruitment was limited to self-identified Somali community members living in Rochester, Minnesota, and the surrounding areas. All study procedures were approved by the Mayo Clinic Institutional Review Board.

### Data collection

The survey was administered by 3 bilingual RHCP community partners and study team members. It was entirely community placed, and various practical approaches were used in recruitment through nonprobability snowball sampling. Possible participants were approached in various social gathering places, including places of worship, sporting events, offices, homes, restaurants, and schools. Besides using existing community events, the community partners also organized small gatherings and meetings solely for recruitment for this study. Data were collected at recruitment or at another prearranged time at the convenience of the study participants.

### Data collection instruments

Data collection instruments were prepared by academic and community partners from the Somali community. Survey items were obtained from existing validated instruments. Most instruments did not previously exist in the Somali language. These items were edited by RHCP partners, adapting the World Health Organization’s translation procedure for use with survey instruments in a CBPR framework [31]. This multistep process includes the following: forward translation, panel discussion, backward translation, pretesting, cognitive briefing, and a consensus on the final instrument by a core group of Somali community partners [32].

### Demographic, biometric, and health behavior measures

Demographic information collected through a survey included the following: age, sex, ethnicity, country of birth, annual household income, education level, employment status, number of years lived in the US, primary language spoken at home, and level of English language proficiency on a 4-point Likert scale.

Weight was measured to the nearest 0.1 kg with a portable digital floor scale. Height was measured to the nearest 0.1 cm with a stadiometer. Body mass index (BMI) was calculated as weight in kilograms divided by height in meters squared. For each participant, data on dietary behaviors were obtained with a dietary screener of 7 items adapted from the food behavior checklist [33]. The food behavior checklist questions include color images of vegetables (fresh and cooked) and fruits that depict a serving size Physical activity was assessed with the International Physical Activity Questionnaire (IPAQ), and calculated per a pre-specified protocol to define three activity levels (https://sites.google.com/site/theipaq/scoring-protocol) [34].

### Network measures and characteristics

#### Structural networks measures

Egocentric, or personal network, data were collected from each survey participant to address the hypotheses of the study. To identify network members, participants were asked to name up to 10 members of their social network (ie, their discussion network). Names and phone numbers were collected for each alter (ie, network member) to verify identities because of the similarity in names among members of the Somali community. For network members without survey data, perceived dietary quality and physical activity level for each named alter were collected from each participant. The weight status of each named alter was described on a 4-point scale (underweight, normal weight, overweight, or very overweight) by the participant who identified the alter as a social tie (ie, connection). For the analysis, the overweight and very overweight groups were consolidated into 1 overweight group. If the network member was named by more than 1 survey respondent, consensus on overweight status was determined from the average of all reports (with 1 point assigned to “underweight” and 4 points to “very overweight”). The average number of overweight network members was calculated for each survey respondent who reported a network tie. The average was calculated as the number of overweight network members divided by the total number of reported ties.

Network size for each participant was calculated as the number of persons with whom a participant had a reported tie.

#### Functional networks measures

For all participants, social norms for obesity and obesity-related behaviors were measured with a 3-item instrument to assess social acceptability of being overweight, eating an unhealthy diet, and being physically inactive, and were reported on a 5-point Likert scale (internal consistency, Cronbach α = 0.69) [35]. Social cohesion was measured with a 3-item measure to assess the “sense of belonging” construct and was reported on a 5-point Likert scale (reliability, Cronbach α = 0.95) [36].

Social support for eating a healthy diet was assessed with a 5-item instrument and reported on a 5-point Likert scale (internal consistency, Cronbach α = 0.61–0.91) [37].

For participants who were affected by obesity and those who were overweight only, the number of social contacts trying to lose weight was assessed on a 5-point scale from 0 (nobody) to 4 (all). Intention to lose weight in the next 3 months was assessed with a 1-item measure (How likely are you to try to lose weight within the next 3 months?) and reported on a 5-point likeness scale. Social norms for weight loss were obtained with a 3-item instrument reported on a 5-point Likert scale (reliability, Cronbach α = 0.70) [35].

In this study, the social cohesion instrument demonstrated excellent internal consistency (Cronbach alpha 0.93) with individual item loading ranging from 0.89 to 0.94. The social norms for obesity instrument demonstrated good internal consistency (Cronbach alpha 0.85) with individual item loading ranging from 0.80 to 0.90. The social support instrument demonstrated excellent internal consistency (Cronbach alpha 0.92) with individual item loading ranging from 0.84 to 0.89. The social norms for weight loss instrument demonstrated fair internal consistency (Cronbach alpha 0.73) with individual item loading ranging from 0.68 to 0.85. On bivariate analysis, social support was correlated with social norms for weight loss (correlation of 0.35, *p* < 0.0001), social cohesion (correlation of 0.22, *p* < 0.0001), and social norms for obesity (correlation of − 0.20, *p* < 0.001). There were no correlations between any of the other three functional network characteristics.

### Peer leader identification

To identify potential peer interventionists, participants were asked to name the people that they go to most often when they need advice, including general advice and specific advice about diet, physical activity, and weight loss. They also named people that they and others respected as leaders in the community.

### Data analysis

Descriptive statistics were used for demographic, biometric, and survey data. Logistic models were incorporated to assess the association between participant and network demographics with binary characteristics such as BMI status and weight loss intention. For estimation of causal mediation effects, the method described by VanderWeele was used [38]. Associations were assessed with logistic models with no adjustments for other covariates. Analyses were performed with SAS version 9.2 software (SAS Institute Inc) and, in particular, the SAS CAUSALMED procedure for mediation analysis.

Network data were analyzed to identify nodes and ties and to visualize the network. The study assessed participants’ egocentric (ie, personal) networks. Network members were linked across individuals through matching names and phone numbers. A hybrid approach, of compiling egocentric network data to form a larger sociocentric network, was used to visualize this real-world community network. If the names and phone numbers of named network members did not match, the members were considered as different individuals in the analysis, so that any error would be an underestimate of position and importance in the overall network. Edgelists of egos and alters were created, and ego attribute data based on survey responses were compiled to import into network analytic software with Stata (StataCorp LLC) [39]. UCINET (Analytic Technologies) [40], Gephi (Gephi Consortium) [41], and R (R Foundation) [42] were used to calculate network metrics (UCINET and Gephi), to conduct more complex network analysis (UCINET and R), and to visualize social networks (Gephi). Although a hybrid approach was used to build and visualize a larger community network, the measures used in the analysis were egocentric in nature (ie, percentage of personal network that is overweight).

## Results

The survey and biometric measurements were completed by 646 participants. This number exceeded the recruitment goal of at least 30% of the Somali adults residing in Rochester, Minnesota, according to the 2007–2011 American Community Survey. Demographic characteristics are shown in the Table 1. The mean (SD) age was 37.5 (16.7) years; 45% of the participants were female; 69% of the participants had a high school education or less; and 75% reported an annual family income of less than $30,000. Most (93%) of the participants were born outside the US, and 65% had limited proficiency with the English language. Participants 50 years or older had lived in the United States longer than younger participants (15.4 years vs 13.2 years, *P* = .04).

**Table 1:** Participant Demographics and Survey Responses (*N* = 646)^a^

Measurement	Female (*N* = 278)	Male (*N* = 364)	Total (*N* = 646)
Age, mean (SD), y	39.4 (16.2)	36.0 (17.0)	37.5 (16.7)
Formal schooling completed, No. (%)			
≤ 8 grades	124 (45)	41 (11)	165 (26)
Some high school	23 (8)	41 (11)	64 (10)
High school or general equivalency diploma	60 (22)	134 (37)	194 (30)
Some college or technical school	49 (18)	98 (27)	147 (23)
College or advanced degree	19 (7)	49 (13)	68 (11)
Annual family income, $, No. (%)			
0–9999	130 (47)	130 (36)	260 (41)
10,000-19,999	49 (18)	53 (15)	102 (16)
20,000-29,000	53 (19)	61 (17)	114 (18)
30,000-49,999	31 (11)	80 (22)	111 (18)
≥ 50,000	12 (4)	34 (9)	46 (7)
Health insurance in past 12 mo, No. (%)			
Yes	263 (95)	313 (86)	576 (90)
No	13 (5)	50 (14)	63 (10)
Country of birth, No. (%)			
United States	14 (5)	31 (9)	45 (7)
Other	255 (595)	319 (91)	574 (93)
English language proficiency, No. (%)			
Not at all	34 (13)	22 (6)	56 (9)
Not very well	94 (35)	59 (16)	153 (24)
Well	72 (26)	121 (34)	193 (31)
Very well	72 (26)	158 (44)	230 (36)
Body mass index, mean (SD)	28.1 (6.1)	25.1 (4.7)	26.4 (5.5)
Normal weight (BMI 18.5 < 25), No. (%)	97 (35)	211 (59)	308 (49)
Overweight (BMI 25 < 30), No. (%)	86 (31)	97 (27)	183 (29)
Obese, No. (%)	92 (33)	52 (14)	144 (23)
Dietary behaviors			
Do you drink regular soda? No. (%)			
No	134 (49)	106 (29)	240 (38)
Sometimes	108 (39)	174 (48)	282 (44)
Often	23 (8)	53 (15)	76 (12)
Every day	11 (4)	29 (8)	40 (6)
Do you drink fruit drinks, punch, or sports drinks? No. (%)			
No	122 (44)	67 (19)	189 (30)
Sometimes	79 (29)	146 (41)	225 (35)
Often	63 (23)	101 (28)	164 (26)
Every day	13 (5)	46 (13)	59 (9)
No. of servings of vegetables daily, mean (SD)	2.2 (1.3)	2.2 (2.1)	2.2 (1.7)
No. of servings of fruit daily, mean (SD)	2.3 (1.3)	2.2 (2.0)	2.2 (1.8)
Physical activity,^b^ No. (%)			
Low	71 (26)	48 (14)	119 (19)
Moderate	109 (40)	104 (29)	213 (34)
High	94 (34)	202 (57)	296 (47)
Self-efficacy for healthy diet, mean (SD)^c^	4.2 (0.9)	3.9 (1.0)	4.0 (0.99)
Self-efficacy for physical activity, mean (SD)^c^	4.1 (1.0)	4.0 (1.0)	4.1 (0.97)
Social support for healthy diet, mean (SD)^d^	3.7 (0.9)	3.2 (0.9)	3.4 (0.9)
Social cohesion, mean (SD)^e^	5.9 (0.8)	5.9 (1.1)	5.9 (1.0)
Social norms for obesogenic behaviors, mean (SD)^d^	2.0 (1.0)	2.4 (1.1)	2.2 (1.1)
Social norms for weight loss, mean (SD)^d^	2.2 (0.8)	2.1 (0.9)	2.2 (0.8)
How likely are you to try to lose weight in the next 3 months? No. (%)			
Very unlikely or unlikely	5 (3)	14 (9)	19 (4)
Neither likely nor unlikely	24 (13)	33 (20)	57 (17)
Very likely or likely	149 (84)	116 (71)	265 (78)

^a^Totals vary because of missing data and combined categories. Gender was missing for 4 participants

^b^International Physical Activity Questionnaire [34]

^c^Reported from a 10-point scale, with 1 being the lowest response option and 10 the highest

^d^Reported from a 5-point Likert scale, with 1 being the lowest response option and 5 the highest

^e^Reported from a 7-point Likert scale, with 1 being the lowest response option and 7 the highest

Half the participants were overweight or affected by obesity, and mean (SD) BMI was 26.4 (5.5). Among participants who were 50 years or older, BMI was significantly higher: mean (SD), 28.3 (5.1); *P* < .001. Among participants with a BMI of at least 25 (*n* = 328), 79% reported an intention to likely or very likely try to lose weight in the subsequent 3 months.

Among all participants, 62% reported drinking regular soda at least sometimes, and 70% reported drinking fruit drinks, punch, or sports drinks at least sometimes. The mean (SD) number of daily servings of vegetables was 2.2 (1.7), and the mean (SD) number for fruit was 2.2 (1.8); 47% of respondents reported high physical activity level on the IPAQ.

### Network characteristics

**Fig. 1:**
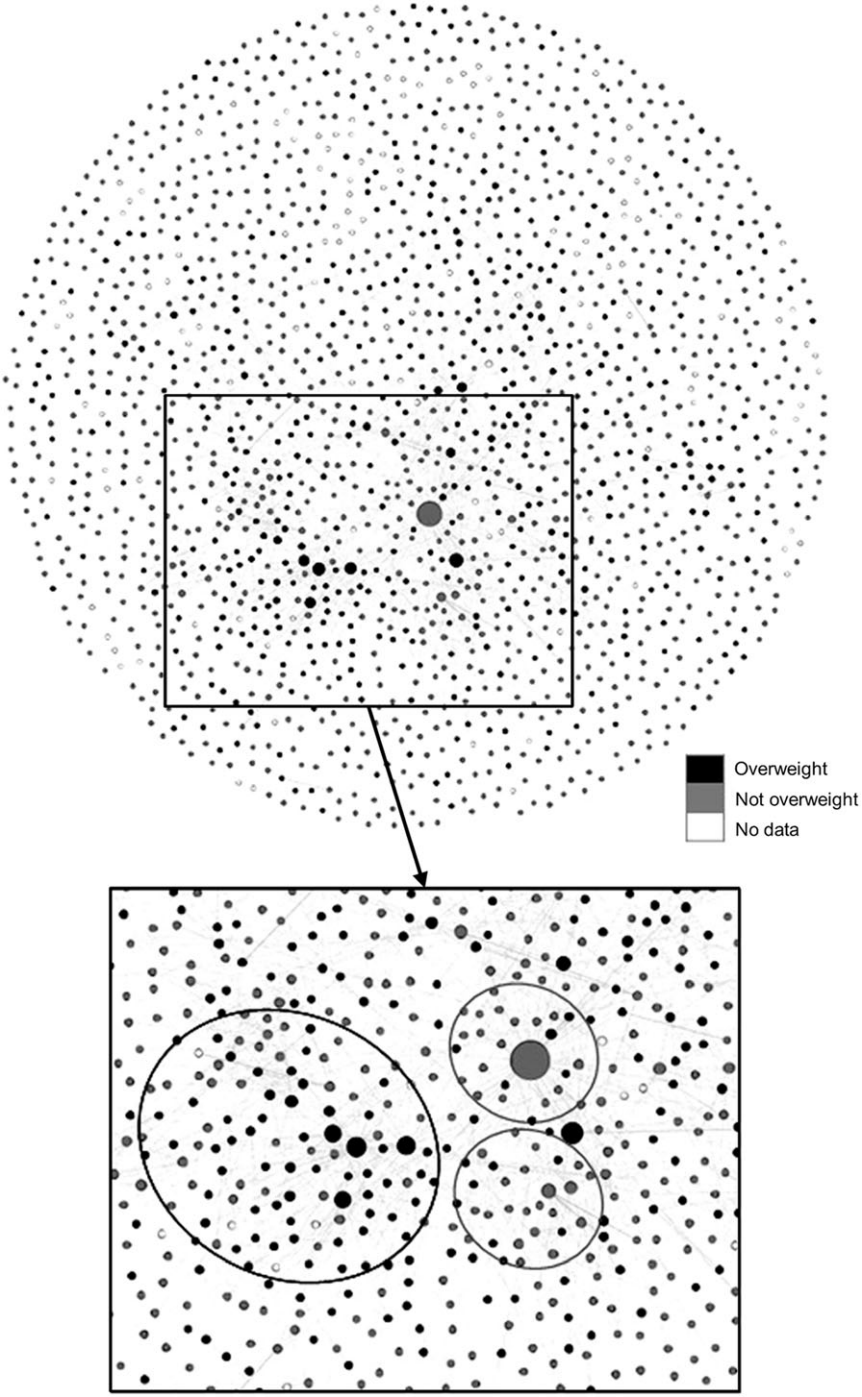
Network According to Weight Status. Nodes are sized on in-degree or number of incoming ties

The network had 1703 nodes with 3583 ties between nodes. Figure 1 shows the network according to weight status. For the entire network sample, including all survey respondents and named alters (*N* = 1703), the percentage of the sample that was reported as overweight or affected by obesity was 26.7%.

Mean (SD) network size was 5.7 (1.3). It was significantly smaller among participants 50 years or older (5.5 [1.2] vs 5.8 [1.3], *P* < .001).

Social norms for obesity among the entire sample were low (mean [SD], 3.6 [3.2]), meaning that there was low acceptability of being overweight or affected by obesity, eating unhealthy food, or being physically inactive among family and friends. Among participants who were overweight or affected by obesity, social norms favoring weight loss were low (mean [SD], 2.2 [0.8]). Social support for healthy diet was high (mean [SD], 3.4 [0.9]). Social cohesion was also high (mean [SD], 5.9 [1.0]). Participants 50 years or older had significantly higher social cohesion (*P* = .045), lower obesogenic social norms (*P* < .001), and higher social support (*P* = .02). There was no significant difference in duration of stay in the United States between participants who were overweight and those who were of normal weight.

### Association between weight status of participants and their social networks

**Fig. 2:**
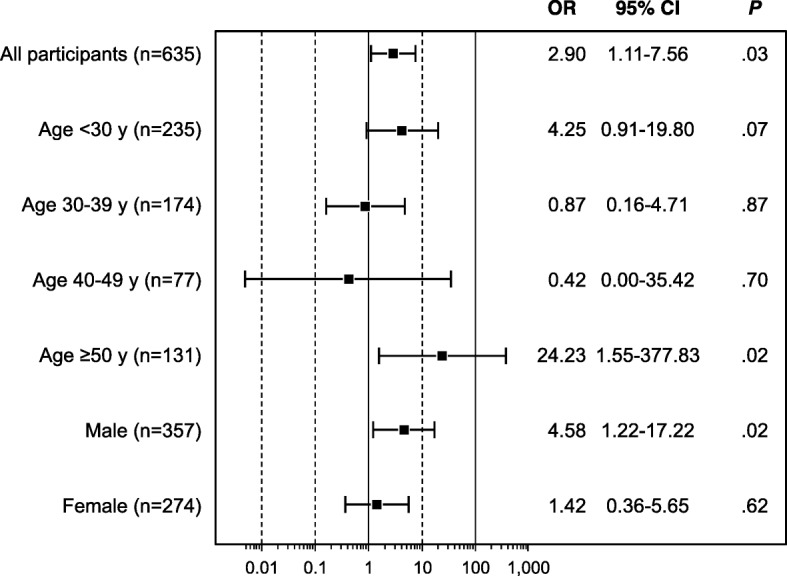
Association Between Participants Being Overweight or Obese and Number of Overweight Network Members. Age and sex are for all participants. Squares indicate odds ratios (ORs). Error bars indicate 95% CIs

Compared with network members of normal weight, overweight or obese members had more network members who were also overweight or obese (odds ratio [OR], 2.90; 95% CI, 1.11–7.56; *P* = .03). This was most notable for those 50 years or older (OR, 24.23; 95% CI, 1.55–377.83; *P* = .03) and for men (OR, 4.58; 95% CI, 1.22–17.22; *P* = .02). Overall findings and results by age and sex are shown in Fig. 2.

Those who were affected by obesity also had more overweight network members than those who were overweight, although this was not statistically significant (14.1% vs 12.4%, *P* = .24).

### Mediation analysis for association between weight status of participants and their social networks

There were no statistically significant mediation effects between participants who were overweight or affected by obesity and the weight status of their social networks. Overweight and affected by obesity participants had a larger mean (SD) network size than participants with normal weight (5.8 [1.1] vs 5.6 [1.4], *P* = .045), but network size did not mediate the relationship between the weight status of participants and their social networks.

The correlation between participant BMI and social norms for obesity was small (Pearson correlation − 0.13, *P* = .001). Likewise, the correlation between overweight social network members and social norms for obesity was small (Pearson correlation 0.21, *P* < .001). However, social norms for obesity did not mediate the total effect of the weight status of the social network members. Similarly, social support and social cohesion did not mediate the relationship between weight status of participants and their social networks.

### Association between weight loss intentions among overweight or participants affected by obesity and social network influences

**Fig. 3:**
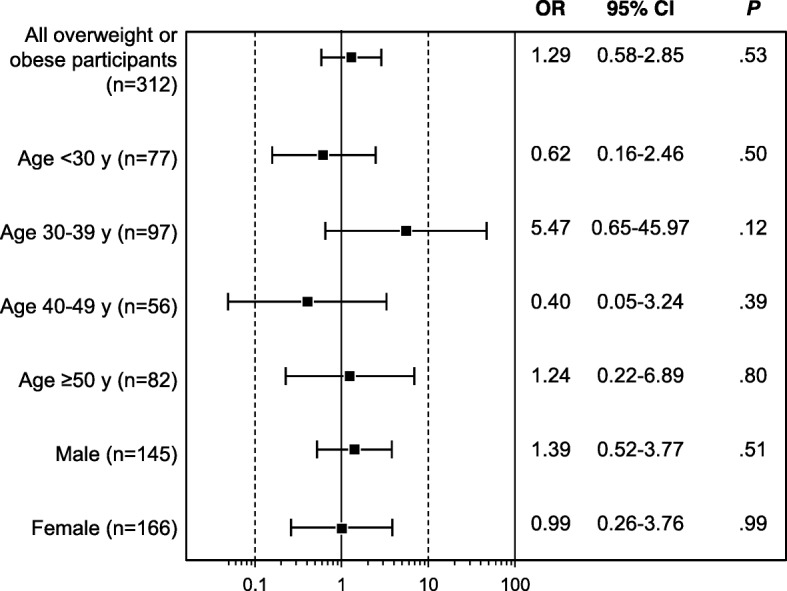
Association Between Overweight or Obese Participants’ Weight Loss Intentions and Network Members’ Weight Loss Intentions. Age and sex are for all overweight or participants affected by obesity. Squares indicate odds ratios (ORs). Error bars indicate 95% CIs

Of the participants who reported that most or all of their family members and friends were trying to lose weight, 38% said that they were very likely to lose weight, compared with 17% of other participants who said that they were very likely to lose weight (*P* = .01). However, there was no significant difference between groups if “likely” and “very likely” responses were combined (83% vs 80%, *P* = .60). There was no significant association between weight loss intentions and the number of social contacts trying to lose weight (OR, 1.29; 95% CI, 0.58–2.85). Overall findings and results by age and sex are shown in Fig. 3.

Participants who stated that they were likely or very likely to lose weight, compared with participants who were not likely to lose weight, reported a slightly larger network size (OR, 1.14; *P* = .047) and significantly more positive (reenforcing) social norms for weight loss (mean [SD], 2.4 [0.75] vs 1.6 [0.67]; *P* < .001); more positive social support for weight loss (mean [SD], 3.4 [0.94] vs 3.1 [0.91]; *P* = .004); less favorable obesogenic social norms (mean [SD], 2.0 [1.03] vs 2.6 [0.94]; *P* < .001); and more social cohesion (mean [SD], 6.0 [0.89] vs 5.7 [1.05]; *P* = .02).

### Mediation analysis for association between weight loss intentions among overweight or participants affected by obesity and social network influences

There was no statistically significant relationship between participants’ likelihood of losing weight and the number of family and friends trying to lose weight; therefore, there were no mediators for this relationship.

The best model for predicting participants’ likelihood to lose weight included social norms for weight loss and obesogenic social norms. Network size was not a mediator of weight loss intention.

### Association between social contacts trying to lose weight among overweight or Participantsaffected by)besity and social network influences

Participants who stated that most or all of their social contacts were trying to lose weight, compared with participants whose social contacts were not trying to lose weight, reported more positive social norms for weight loss (mean [SD], 2.9 [0.7] vs 2.1 [0.7]; *P* < .001); more positive social support for eating a healthy diet (mean [SD], 3.6 [0.93] vs 3.3 [0.94]; *P* = .03); and more moderate to high levels of physical activity (88.0% vs 70.6%, *P* = .003).

## Discussion

To our knowledge, this is the first social network analysis study of obesity and related health behaviors among Somali immigrants in the US. Findings from this study indicate that this community has discernible social networks, which are clustered by weight status. The robust and successful recruitment of participants for this study, from a group that has been seen as traditionally difficult for researchers to reach, lends credence to the community-based participatory research approach and holds promise for future intervention work on obesity with the Somali community.

Our study identified clustering of social networks by body weight status, which is consistent with the plurality of studies of general populations [18, 43], thereby extending this association to the Somali immigrant communities in Minnesota. This clustering was most pronounced among men and participants 50 years or older. In the present study, older participants had only slightly smaller networks, had lived in the United States longer, and had significantly higher social cohesion and social support for eating a healthy diet. Previous research showed that older immigrants tend to exhibit lower acculturation than younger groups [44]. Acculturation can be conceived as a multifaceted process of integration on the part of immigrants and their receiving communities [45]. Lower acculturation has been associated with lower rates of obesity and cardiovascular risk among African immigrants [46–48]. Results from our study imply that social cohesion and social support, which are associated with healthy behaviors for obesity [49, 50], may be important in weight control among older Somali adults.

Weight loss intention, an important prerequisite for actual weight loss, is positively associated with successful weight loss [51]. In our study, the overweight or participants affected by obesity who reported the highest degree of weight loss intention were the ones who stated that most or all of their family and friends were trying to lose weight. Furthermore, those participants’ weight loss intentions were associated with more favorable social norms for weight loss and with higher social support and social cohesion. These findings are consistent with previous studies of general populations that found that weight loss intentions may be influenced by enabling functional network factors, especially social support and social norms for weight loss [52, 53]. At the same time, social support and social norms for weight loss are key factors in facilitating weight loss, and it has been proposed that incorporating them in traditional weight loss interventions may lead to more success [52, 54].

In the present study, among participants who were overweight or affected by obesity, an association existed between the number of social contacts trying to lose weight and the social norms for weight loss, social support, and activity level. This finding is consistent with the knowledge that people’s behavior may be influenced by social ties and contacts [52, 55]. Our results extend this finding to the Somali community in Minnesota and imply that these social ties could be harnessed to promote weight loss [56]. Interestingly, among overweight or participants affected by obesity, there was no association between the number of social contacts trying to lose weight and social cohesion, which has been associated with health-influencing behaviors among and within groups [57]. This finding may be explained by the low number of social contacts trying to lose weight, especially because weight loss intention was associated with social cohesion, while perception of being physically inactive was inversely associated with social cohesion.

The strengths of the study are that it used a large, community-based sample of a Somali immigrant population. The community-based participatory research approach was key to successful participant recruitment and participation in the study. The study had several limitations: It was confined to 1 city, and thus the results may not be geographically generalizable. The use of perceived weight status for alters was prone to underreporting bias, which likely led to weaker measured associations between weight status and other network characteristics. Likewise, BMI was used as a sole objective classification scheme for assessment of weight class. Finally, the cross-sectional nature of the study and possibility of other unmeasured confounding factors, limits drawing conclusions to associations only and not causality.

## Conclusion

Overall, the findings from this study show that distinct social networks in this community are clustered by weight status and that social contacts and functional network characteristics are related to weight loss intentions. These factors should be considered in weight loss interventions and programs. A social network intervention for weight loss in Somali communities, within a CBPR framework, seems plausible.

## Data Availability

The datasets generated and/or analyzed during the current study are available from the corresponding author on reasonable request.

## Abbreviations

BMI: Body mass index
CBPR: Community-based participatory research
HIF: Healthy Immigrant Families
IPAQ: International Physical Activity Questionnaire
OR: Odds ratio
RHCP: Rochester Healthy Community Partnership
US: United States
